# A Case of Asymptomatic Carotid Artery Stenosis With Thumb‐Sized Calcification Detected on Panoramic Radiograph

**DOI:** 10.1002/ccr3.73620

**Published:** 2026-11-01

**Authors:** Kosei Shibata, Keiko Kaneko, Tatsuo Takaya, Toshiyuki Inou, Yu Chi Hung, Hiroyuki Kitamura, Emi Oki, Hideyuki Nezu, Toshiaki Ara, Shunsuke Funaoka, Daisuke Higuchi, Nanae Dewake, Masahiro Okuda, Mitsuji Muneyasu, Nobuo Yoshinari, Keiichi Uchida

**Affiliations:** ^1^ Department of Prosthodontics, School of Dentistry Matsumoto Dental University Shiojiri Japan; ^2^ Department of Oral Diagnostics and Comprehensive Dentistry Matsumoto Dental University Hospital Shiojiri Japan; ^3^ Department of Periodontology, School of Dentistry Matsumoto Dental University Shiojiri Japan; ^4^ Department of Pharmacology, School of Dentistry Matsumoto Dental University Shiojiri Japan; ^5^ Faculty of Science and Engineering Doshisha University Kyoto Japan; ^6^ Kansai University Osaka Japan

**Keywords:** carotid artery diseases, carotid stenosis, chronic kidney failure, panoramic radiography, ultrasonography

## Abstract

A 73‐year‐old male with dyslipidemia and chronic kidney disease exhibited carotid calcification on a dental panoramic radiograph. Ultrasonography confirmed 30% asymptomatic carotid stenosis. This case highlights intimal calcification's link to kidney disease and atherosclerosis. Prompt medical‐dental collaboration and diagnostic imaging remain crucial to prevent cerebrovascular events, extending healthy life expectancy.

## Key Clinical Message

Routine panoramic radiographs can serve as a vital window for identifying asymptomatic carotid artery calcification. Dentists should be vigilant for unusually large cervical radiopacities, particularly in patients with chronic kidney disease, and promptly initiate medical–dental collaboration to prevent life‐threatening cerebrovascular events.

## 1 Introduction

Atherosclerotic carotid artery stenosis is a progressive, age‐related condition exacerbated by lifestyle‐related risk factors, such as hypertension, diabetes, and hyperlipidemia [1]. In dentistry, calcifications suggestive of carotid artery stenosis are occasionally identified on panoramic radiographs as opacities in the anterior carotid artery course between the third and fourth cervical vertebrae. Along with the coronary and lower limb arteries, the carotid artery is particularly prone to atherosclerosis [2], and these calcifications represent atherosclerotic lesions that may lead to vascular complications. In recent years, with the widespread use of brain health screenings and advances in diagnostic imaging technology, such calcifications are increasingly detected in asymptomatic patients. In this report, we describe a case of relatively large carotid artery calcifications identified on a panoramic radiograph, together with corresponding imaging findings.

## 2 Case History/Examination

A 73‐year‐old male presented to the Initial Consultation Office (Department of General Diagnosis and General Treatment) at Matsumoto Dental University Hospital with gingival swelling in the left mandibular first molar region. His medical history included dyslipidemia and chronic kidney disease (CKD), for which he had received long‐term care at the university's Department of Internal Medicine over several years. He attends regular follow‐up appointments and takes oral medications, including rosuvastatin (2.5 mg once daily; Crestor, AstraZeneca, Cambridge, UK), pemafibrate (0.1 mg twice daily; Parmodia, Kowa Company Ltd., Nagoya, Japan), and dapagliflozin (5 mg once daily; Farxiga/Forxiga, AstraZeneca, Cambridge, UK), under ongoing supervision at a primary care internal medicine clinic.

At presentation, intraoral examination revealed periodontal pockets exceeding 4 mm with gingival bleeding in the bilateral mandibular first molars and bilateral maxillary first and second molars. Because periodontal disease was suspected, a panoramic X‐ray was obtained. After being informed that the findings suggested atherosclerotic lesions (Figure 1A–C), the patient reported no prior imaging evaluation for atherosclerosis, such as ultrasound or magnetic resonance angiography. Therefore, the treating dentist referred him to the Department of Internal Medicine at our university for carotid ultrasonography.

**FIGURE 1:**
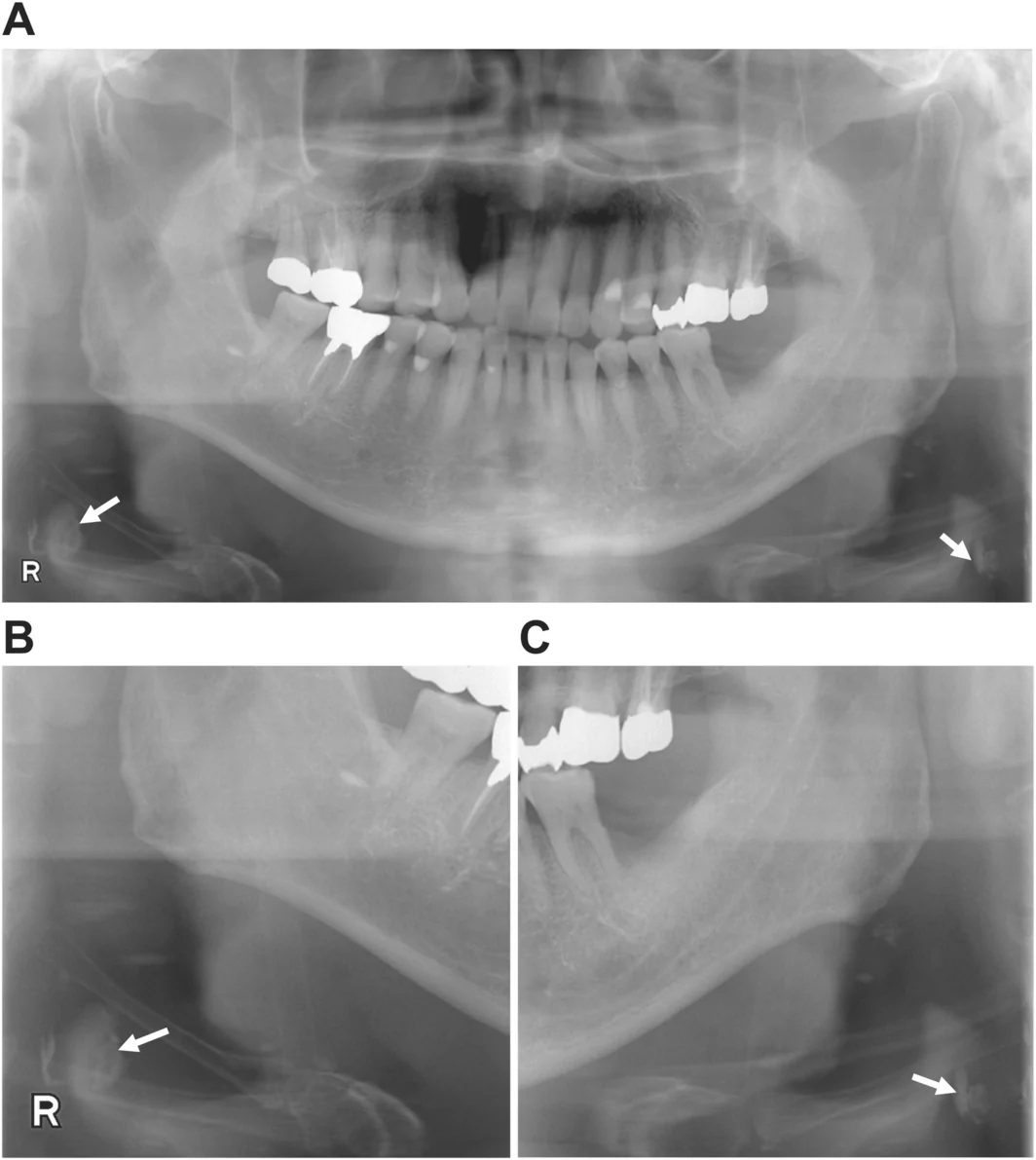
Panoramic radiograph demonstrating carotid artery calcification. (A–C) Panoramic images revealed a thumb‐sized calcification with well‐defined borders and a slightly heterogeneous interior in the right cervical region, along with scattered calcifications surrounding it (arrows). Scattered, irregularly shaped calcifications were also observed in the left cervical region (arrows).

## 3 Differential Diagnosis, Investigations, and Treatment

Carotid ultrasound identified a homogeneous plaque measuring 4.0 × 30.6 × 3.8 mm in the right common carotid artery. In the right carotid bulb, multiple heterogeneous calcified plaques were detected, with the largest measuring 11.0 × 3.3 mm, along with a homogeneous calcified plaque measuring 6.0 × 2.0 mm in the right internal carotid artery (Figure 2A–D). On the left side, a homogeneous plaque measuring 1.61 × 3.8 mm was observed in the common carotid artery, and multiple heterogeneous calcified plaques were present at the carotid bifurcation, measuring 9.9 × 3.9 mm, 16.9 × 4.0 mm, and 11.3 × 30.0 mm (Figure 3A–D). Based on these findings, the patient was diagnosed with carotid artery stenosis. Multiple conditions can present as cervical radiopacities near the neck on panoramic imaging. Differential diagnoses include stylohyoid ligament ossification, salivary stones, calcified cervical lymph nodes, venous stones, and anatomical overlap of the hyoid bone.

**FIGURE 2:**
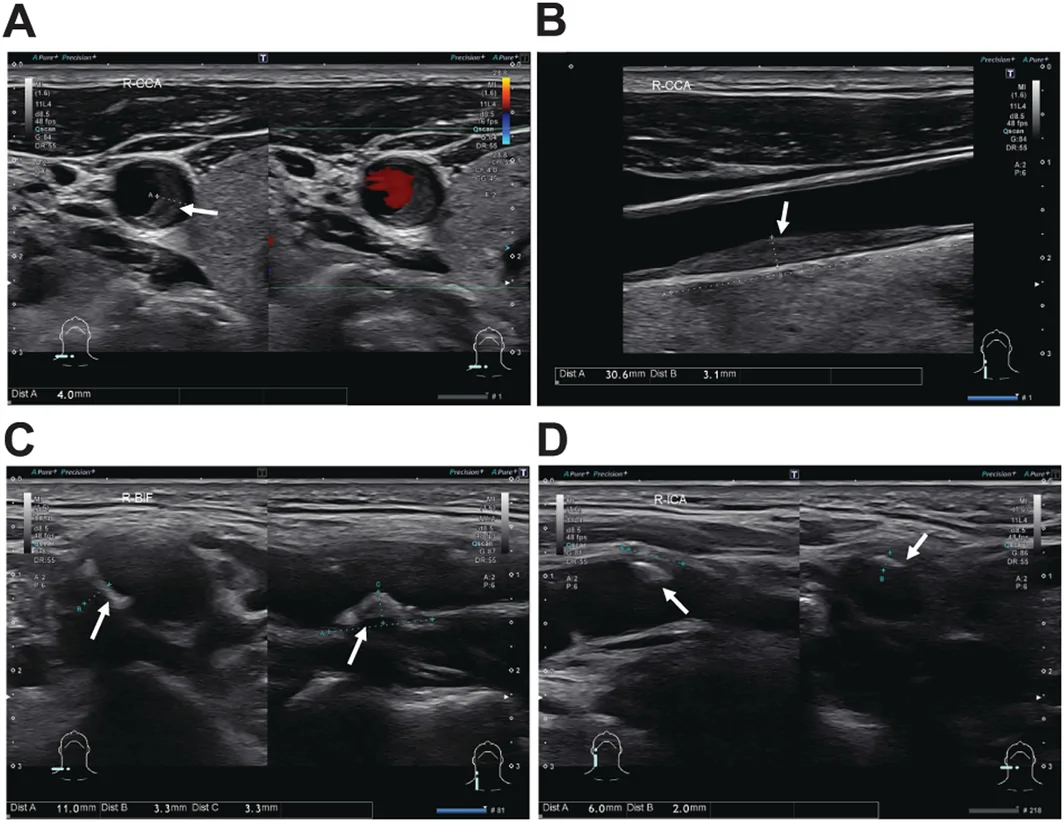
Carotid ultrasonography findings in the right carotid artery. (A) Carotid ultrasound image of the right common carotid artery (R CCA) showing a homogeneous plaque (arrow) measuring 4.0 × 30.6 × 3.8 mm. The accompanying color Doppler image indicates the presence of blood flow. (B) Longitudinal view of the R CCA further illustrating the plaque (arrow), measuring 30.6 mm in length. (C) Ultrasound image of the right carotid bulb (R BIF) revealing multiple heterogeneous calcified plaques, the largest measuring 11.0 × 3.3 mm (arrows). (D) Ultrasound image of the right internal carotid artery (R ICA) showing a homogeneous calcified plaque measuring 6.0 × 2.0 mm (arrows).

**FIGURE 3:**
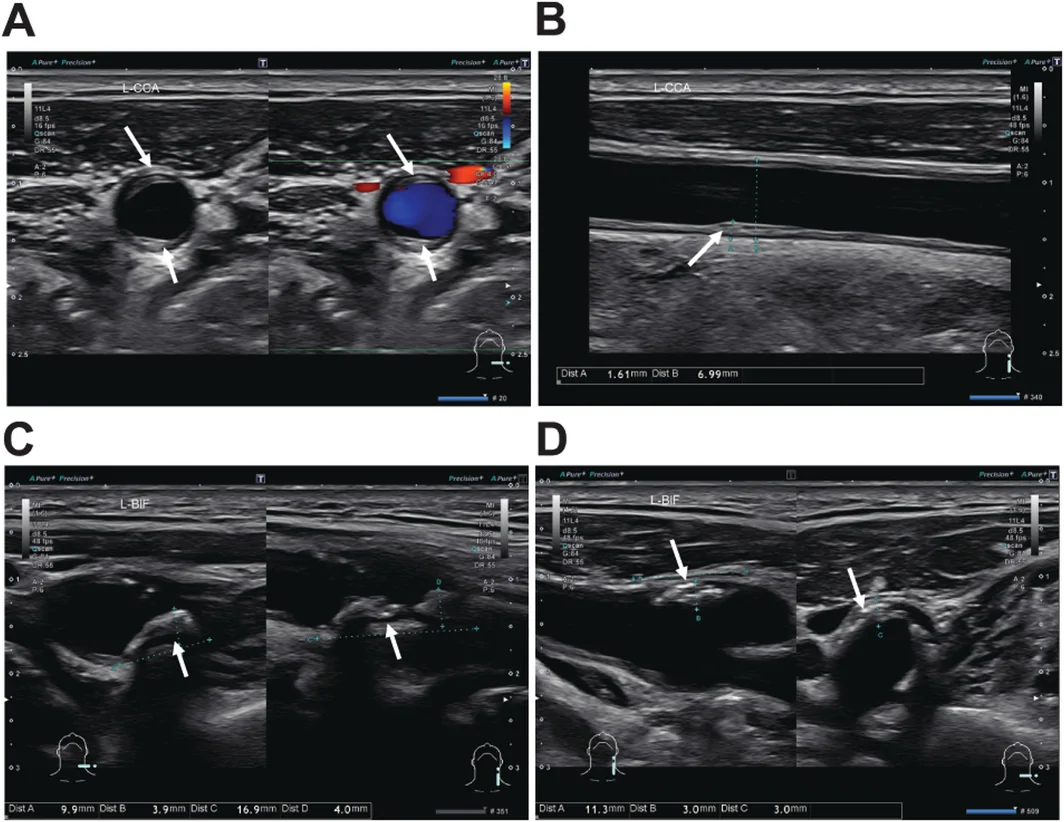
Carotid ultrasonography findings in the left carotid artery. (A) Ultrasound image of the left common carotid artery (L CCA) showing a homogeneous plaque (arrows) measuring 1.61 × 3.8 mm. The accompanying color Doppler image indicates the presence of blood flow. (B) Longitudinal view of the L CCA demonstrating the plaque (arrow), measuring 1.61 mm in height. (C) Ultrasound image of the left carotid bifurcation (L BIF) displaying multiple heterogeneous calcified plaques, the largest measuring 9.9 × 3.9 mm (arrows). (D) Another view of the L BIF illustrating additional plaques, including one measuring 11.3 × 30.0 mm (arrows) and another measuring 16.9 × 4.0 mm.

## 4 Conclusion and Results (Outcome and Follow‐Up)

Because the degree of stenosis was approximately 30%, the Department of Internal Medicine elected conservative management with ongoing medical therapy and periodic carotid ultrasound monitoring. Concurrently, strict control of underlying conditions, including dyslipidemia and CKD, is being maintained. The clinical course of the patient is summarized in Figure 4.

**FIGURE 4:**
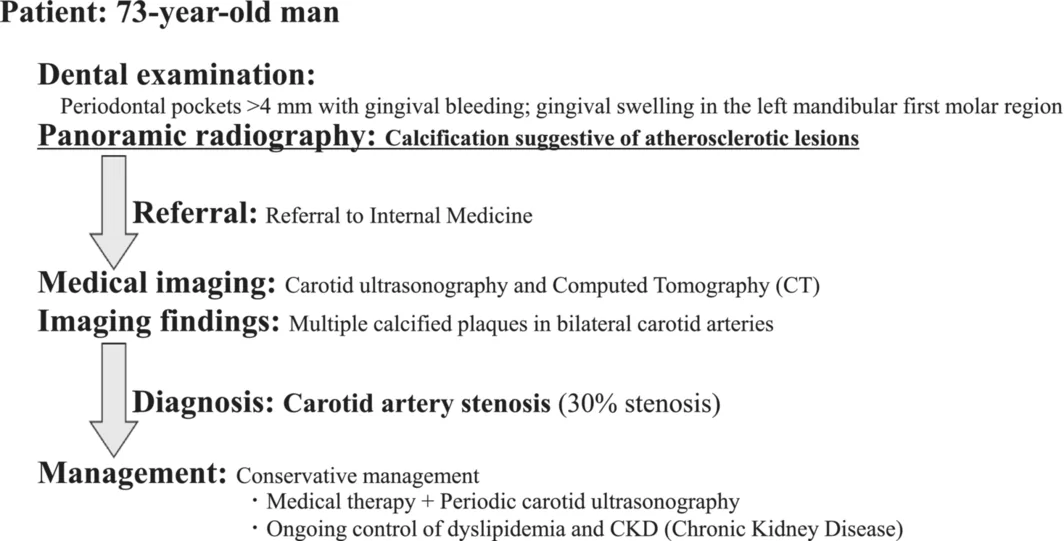
Clinical course of the patient. A 73‐year‐old man presented with periodontal disease, and panoramic radiography revealed carotid calcification suggestive of atherosclerotic lesions. Subsequent carotid ultrasonography and computed tomography confirmed bilateral calcified plaques with 30% carotid artery stenosis, and the patient was managed conservatively with medical therapy and periodic follow‐up.

## 5 Discussion

Carotid artery stenosis is a progressive condition in which atherosclerotic plaque develops within the carotid intima, undergoes calcification, and leads to luminal narrowing; it occurs most commonly at the bifurcation of the common carotid artery [3]. Atherosclerosis arises from lipid deposition in the vascular wall with subsequent infiltration and accumulation of macrophages that have phagocytosed these lipids [4].

On panoramic X‐ray images obtained in dental practice, portions of the carotid artery adjacent to the oral cavity may be visualized, and calcifications can occasionally be detected. Carotid artery calcification on panoramic radiographs was first reported by Friedlander [5]. The carotid bifurcation between the third and fourth cervical vertebrae is a typical site, appearing as nodular to irregular opacities anterior to these vertebrae and posteroinferior to the mandibular angle. The common carotid artery branches into the internal and external carotid arteries in the central neck, generally below the mandibular angle. The common carotid artery has been reported to bifurcate into the internal and external carotid arteries at the C3 vertebral level (27.2%), C3/C4 intervertebral level (26.2%), C4 vertebral level (25.5%), and C2/C3 intervertebral level (7.5%), with most bifurcations occurring around the C3–C4 level [6]. Because the third and fourth cervical vertebrae are frequently captured on panoramic X‐ray images, asymptomatic carotid stenosis in this region may be incidentally identified. In the present case, a nodular radiopacity distinct from the hyoid bone was observed anterior to the fourth cervical vertebra and posteroinferior to the mandibular angle. As carotid artery calcification was confirmed via ultrasound and computed tomography, the radiographic finding was considered carotid artery calcification based on its morphology and location. Although prompt medical–dental collaboration occurred in this case owing to the treating dentist's knowledge and experience of carotid artery calcification, accurate interpretation of panoramic X‐ray images requires familiarity with the spatial relationship between carotid artery calcifications and adjacent anatomical structures, as well as the diagnostic acumen to avoid misclassifying such findings as “normal” without appropriate evaluation.

The morphology and size of carotid calcifications in carotid stenosis vary widely, ranging from nodular lesions (the size of a rice grain, a little finger, or a thumb tip) to larger, scattered, or bead‐like deposits. In the current case, ultrasound revealed plaques measuring 30.6 × 3.8 mm in the right common carotid artery, up to 11.0 × 3.3 mm in the right carotid bulb, and 6.0 × 2.0 mm in the right internal carotid artery. These measurements correspond to the radiopaque findings on the panoramic imaging and can be clinically described as relatively large calcifications exceeding thumb‐tip size. Although reports focusing specifically on calcification “size” in carotid stenosis are limited, this present case demonstrates large calcifications compared with routine clinical observations and prior cases. Established risk factors for atherosclerosis include aging, obesity, smoking, physical inactivity, diabetes, dyslipidemia, hypertension, oxidative stress, and genetic predisposition. These factors contribute to a loss of elasticity in the vascular wall, followed by endothelial dysfunction, promoting intima–media complex thickening and progressive plaque formation that may culminate in stenosis or occlusion [4, 7]. Notably, in addition to dyslipidemia, the patient in the current case also had a history of CKD, which is associated with calcium–phosphorus metabolic abnormalities that promote hyperphosphatemia and hypercalcemia, thereby predisposing not only the intima but also the media to ectopic calcification (Mönckeberg‐type calcification) [8]. Although typical atherosclerotic plaques primarily involve intimal thickening, CKD‐associated medial calcification is characterized by extensive, continuous calcium deposition along the vascular wall [9]. The large calcifications observed in the present case likely reflect superimposed medial calcification on intimal atherosclerosis, increasing radiographic opacity and resulting in larger, clearer images.

Accurate interpretation of panoramic radiographs, routinely obtained during dental examinations, is essential for detecting carotid artery calcification and enabling appropriate screening. Asymptomatic carotid stenosis often progresses without noticeable symptoms and represents a major risk factor for cerebral infarction and transient ischemic attacks [10]. Prior studies have indicated that appropriate medical intervention and drug therapy can substantially reduce stroke risk in patients with asymptomatic carotid stenosis [11], underscoring the importance of early detection. Almog et al. [12] reported that sides exhibiting calcification near the carotid bifurcation on panoramic radiographs had a higher prevalence of clinically significant carotid artery stenosis detected by duplex ultrasound (DUS) than sides without calcification. Ultrasound is a noninvasive and highly informative modality that allows for the assessment of stenosis severity, plaque echogenicity, surface characteristics, and hemodynamic parameters, such as peak blood flow velocity at the stenosis site [13]. However, Yoon et al. [14] demonstrated that panoramic radiography has limited but clinically relevant accuracy for detecting carotid artery calcification when compared with computed tomography. Taken together, these findings suggest that when calcifications suggestive of carotid artery stenosis are identified on panoramic radiographs, prompt referral for carotid ultrasonography should be considered, with computed tomography used for further evaluation when clinically indicated.

Future dental care should extend beyond local oral management to incorporate panoramic radiography as a screening tool for systemic disease, supported by close medical–dental collaboration involving internists. Identifying systemic atherosclerotic changes through oral imaging and facilitating early treatment represents an important “predisease intervention” that may help extend healthy life expectancy and maintain quality of life.

This case demonstrates that routine interpretation of panoramic radiographs in dental practice can effectively aid in screening for asymptomatic carotid stenosis. In patients with a history of CKD, ectopic vascular calcification may produce large calcification patterns, requiring detailed anatomical knowledge and strong diagnostic judgment. Early detection of carotid artery calcification by dentists on panoramic images, followed by prompt referral to medical specialists, is crucial for preventing serious cardiovascular events, such as stroke. Strengthening medical–dental collaboration is expected to further support improved patient outcomes and extend healthy life expectancy.

## Author Contributions

**Kosei Shibata:** conceptualization, writing – original draft. **Keiko Kaneko:** conceptualization, supervision. **Tatsuo Takaya:** resources, visualization. **Toshiyuki Inou:** resources, visualization, validation. **Yu Chi Hung:** visualization, resources. **Hiroyuki Kitamura:** resources, visualization. **Emi Oki:** resources, project administration. **Hideyuki Nezu:** writing – review and editing. **Toshiaki Ara:** software. **Shunsuke Funaoka:** writing – review and editing. **Daisuke Higuchi:** writing – review and editing, validation. **Nanae Dewake:** writing – review and editing. **Masahiro Okuda:** project administration, validation. **Mitsuji Muneyasu:** project administration, validation. **Nobuo Yoshinari:** writing – review and editing. **Keiichi Uchida:** conceptualization, supervision, writing – original draft.

## Funding

The authors have nothing to report.

## Consent

In accordance with the journal's patient consent policy, written informed consent was obtained from the patient for the publication of this case report.

## Conflicts of Interest

The authors declare no conflicts of interest.

## Data Availability

The data supporting the findings of this study are available on request from the corresponding author. The data are not publicly available due to privacy or ethical restrictions.
